# Carbapenem Resistance and ESBL-Producing Enterobacteriaceae in Patients with Urological Infections from 2012 to 2021 in Three Korean Hospitals

**DOI:** 10.3390/diagnostics15162004

**Published:** 2025-08-11

**Authors:** Seon Beom Jo, Sun Tae Ahn, Hyung Joon Joo, Jong Wook Kim, Mi Mi Oh

**Affiliations:** 1 Department of Urology, Korea University Guro Hospital, Seoul 08308, Republic of Korea; 2 Department of Cardiology, Korea University Anam Hospital, Seoul 02841, Republic of Korea

**Keywords:** antibiotic resistance, antibiotic stewardship, multidrug-resistant organisms (MDRO), antimicrobial therapy, infection control

## Abstract

**Background:** Urinary tract infections (UTIs) remain a leading cause of community- and hospital-onset bacterial infections worldwide. Although many countries have implemented antimicrobial resistance (AMR) surveillance systems, longitudinal multicenter data on key uropathogens in Korea remain limited. **Methods:** We retrospectively evaluated *Escherichia coli* and *Klebsiella pneumoniae* isolates from patients with clinically diagnosed UTIs at three tertiary-care Korean hospitals (2012–2021). Using a harmonized Observational Medical Outcomes Partnership Common Data Model (OMOP CDM), we analyzed antibiotic susceptibility based on Clinical and Laboratory Standards Institute breakpoints. Trends in resistance to key antibiotics (including fluoroquinolones, cephalosporins, and carbapenems) were assessed using the Cochran–Armitage test. **Results:** From 2012 to 2021, ESBL-producing *E. coli* and *K. pneumoniae* increased from 24.1% to 38.2% and 39.2% to 46.4%, respectively. The rates for *K. pneumoniae* remained stable over the last 6 years, and for *E. coli*, they remained stable over the last 3 years. Resistance rates for *E. coli* increased from 44.5% to 60.0% (ciprofloxacin) and from 26.3% to 40.2% (cefotaxime), while carbapenem resistance (ertapenem) remained low, at 0.3% to 1.2%. In contrast, *K. pneumoniae* exhibited high resistance levels to fluoroquinolones, cephalosporins, and other broad-spectrum antibiotics, with notable increases in resistance to ertapenem, from 3.0% to 18.1%, and imipenem, from 0.4% to 16.8%. This escalation mainly stemmed from the rise in ertapenem (6.6% to 17.0%) and imipenem (0.8% to 14.6%) resistance rates among *Klebsiella*-ESBL producers. **Conclusions:** We conclude that in Korea, the proportion of ESBL-producing *E. coli* and *K. pneumoniae* increased significantly from 2012 to 2018 and has since remained stable for the last 3 years (*E. coli*) and 6 years (*K. pneumoniae*). Although carbapenem resistance in *E. coli* remains low, *K. pneumoniae* has experienced a significant rise, primarily attributable to its ESBL-producing strains. These findings underscore the importance of vigilant antimicrobial stewardship and continuous surveillance to guide empirical UTI therapies in Korean clinical practice.

## 1. Introduction

Urinary tract infections (UTIs) are the most common community-onset bacterial infections worldwide, with substantial implications for public health and healthcare resource utilization [1,2]. In fact, UTIs account for an estimated 150 million cases globally each year, leading to significant direct and indirect costs [3]. The indiscriminate use of antibiotics in treating these infections has led to reduced cure rates, prolonged hospital stays, escalated relapse rates, and accelerated the emergence of antibiotic resistance [4,5,6,7]. Furthermore, multiple international guidelines emphasize the importance of selecting empirical treatments based on local resistance data, as the overuse or misuse of broad-spectrum antibiotics accelerates the development of resistance [6,8]. Antimicrobial resistance (AMR) has become a pressing global health issue, complicating the empirical selection of antibiotics and thereby undermining effective infection management strategies. In 2019, bacterial AMR was directly responsible for approximately 1.27 million deaths and contributed to an estimated 4.95 million deaths globally, underscoring the urgent need for strategies to preserve the efficacy of existing antimicrobial agents [9]. This alarming trend further underscores the global challenge of combating antibiotic resistance and ensuring the efficacy of treatments for bacterial infections, such as UTIs, both locally and globally [9]. Among the most concerning developments is the rise of extended-spectrum β-lactamase-producing Enterobacteriaceae (ESBL-PE), notably *Escherichia coli* and *Klebsiella pneumoniae*, which severely limit treatment options due to their resistance against both β-lactam and non-β-lactam antimicrobials [10,11,12,13]. Recognizing these challenges, many countries, including Korea, have implemented surveillance systems aligned with WHO standards to monitor antibiotic resistance trends. In Korea, the Korean Global Antimicrobial Resistance Surveillance System (Kor-GLASS) provides timely data on key uropathogens such as *E. coli* and *K. pneumoniae*, revealing that fluoroquinolone resistance currently exceeds 20% and is steadily increasing, while trimethoprim-sulfamethoxazole (TMP/SMX) resistance, though still over 40%, shows signs of a downward trend [14,15].

Local epidemiology can differ substantially from national or international averages. Hence, we aimed to examine the 10-year (2012–2021) trend of antibiotic resistance in *E. coli* and *K. pneumoniae* isolates collected from three tertiary-care teaching hospitals in Korea. Using the Observational Medical Outcomes Partnership Common Data Model (OMOP CDM) framework, we harmonized microbiological and clinical data, facilitating standardized analyses of resistance to commonly tested antibiotics, including fluoroquinolones, cephalosporins, carbapenems, and others. We also assessed the prevalence of ESBL production and explored trends in carbapenem resistance within ESBL-producing strains. Our findings may inform local empirical treatment guidelines and stewardship programs while contributing valuable multicenter data to the broader national and global discourse on AMR.

## 2. Methods

### 2.1. Study Design and Setting

This retrospective observational study investigated resistance trends in *Escherichia coli* and *Klebsiella pneumoniae* from 2012 to 2021 at three tertiary-care teaching hospitals—Anam, Guro, and Ansan—affiliated with Korea University. Because patient-level demographic data (e.g., age, gender, department) were not included in our IRB approval (No. 2022GR0467), only aggregate, de-identified data were used for analysis. Each institution maintains an electronic medical record (EMR) system and a laboratory information system (LIS). The study protocol was approved by the Korea University Institutional Review Board (No. 2022GR0467), with a waiver of informed consent due to the retrospective design and use of anonymized data.

### 2.2. Data Harmonization Using OMOP CDM

An extract-transform-load (ETL) process was performed to standardize data from the three centers. We systematically mapped diagnostic codes to the International Classification of Diseases (ICD), and laboratory tests (including antibiotic minimum inhibitory concentration [MIC] results) to Logical Observation Identifiers Names and Codes (LOINC). These mappings were organized under the OMOP Common Data Model (CDM, version 5.3). All personal identifiers were removed or replaced with de-identified keys in accordance with IRB requirements. By using a unified CDM-based structure, we minimized discrepancies among the hospitals and enabled reproducible analyses of resistance trends.

### 2.3. Case Definition and Data Extraction

We identified patients using UTI-relevant diagnostic codes (cystitis N30.0–N30.9, N33.0; prostatitis N41.0–N41.9; and pyelonephritis or upper UTI N10, N28.8, N11.9, N12) from ICD-10. We included all urine culture orders that yielded *E. coli* or *K. pneumoniae*, specifically “Culture & Antibiotic MIC (urinary)” [L0023347] and its historical equivalents [L002224/L002255]. (These order codes refer to internal hospital catalog numbers used in prescription and laboratory information systems.) We excluded records that lacked valid antibiotic susceptibility results or showed ambiguous UTI coding (e.g., incomplete mapping to a confirmed UTI episode). Due to varying documentation across institutions, we were unable to consistently classify UTI types (uncomplicated vs. complicated) or catheter-associated UTIs in all three centers.

### 2.4. Antibiotic Susceptibility Testing

Across the three hospitals, antibiotic susceptibility testing was routinely conducted using the Vitek automated system (bioMérieux, Marcy-l’Étoile, France), employing Clinical and Laboratory Standards Institute (CLSI) guidelines for interpretive categories (susceptible, intermediate, resistant). For Enterobacteriaceae, including *Escherichia coli* and *Klebsiella pneumoniae*, our institutional Vitek panel was configured to test amikacin, amoxicillin-clavulanate, ampicillin, aztreonam, cefazolin, cefepime, cefotaxime, cefoxitin, ceftazidime, ciprofloxacin, ertapenem, gentamicin, imipenem, piperacillin-tazobactam, tigecycline, and trimethoprim/sulfamethoxazole. CLSI guidelines were followed for interpretive criteria, categorizing isolates as susceptible, intermediate, or resistant based on established breakpoint values.

### 2.5. ESBL Detection

Extended-spectrum β-lactamase (ESBL) production in *E. coli* and *K. pneumoniae* was identified using phenotypic synergy tests (comparing MICs with and without clavulanic acid), following CLSI guidelines. Any isolate demonstrating a ≥3 twofold dilution decrease in MIC for cefotaxime or ceftazidime (with clavulanic acid) was designated as ESBL-positive. Molecular typing was not routinely performed, which we note as a limitation.

### 2.6. Panel Exclusions

Certain antibiotics—namely ampicillin-sulbactam, levofloxacin, ceftriaxone, meropenem, and piperacillin—were tested inconsistently across different years or hospitals, resulting in incomplete longitudinal data. Consequently, these agents were excluded from the final dataset to maintain uniformity in resistance trend comparisons. We acknowledge that these antibiotics (e.g., ceftriaxone, meropenem) are used clinically, but the inconsistent testing precluded full inclusion in our 10-year trend analyses. Hence, we focused on cefotaxime and imipenem/ertapenem as the representative third-generation cephalosporin and carbapenems, respectively.

### 2.7. Outcomes and Statistical Analysis

Yearly resistance rates (“%(n/N)”) for each antibiotic were compiled from 2012 to 2021. We used the Cochran–Armitage test for trend (SAS 9.4) to determine whether the proportion of resistant isolates shifted significantly over time. Significance was defined at an α level of 0.05. R (version 4.0.3) was used for data wrangling and descriptive analyses. We then conducted two post-hoc sensitivity analyses: (a) pre-COVID (2012–2019) vs. COVID-period (2020–2021) and (b) exclusion of the earliest two years (2012–2013) to confirm robustness. Hospital-specific 2021 proportions for *Escherichia coli* and *Klebsiella pneumoniae* were compared across the three centers using 3 × 2 χ^2^ tests; 95% CIs were computed with the Wilson method.

### 2.8. Ethical Considerations

All procedures were conducted in accordance with the Declaration of Helsinki and institutional regulations. This study complied with all applicable ethical guidelines and was approved by the Korea University IRB (No. 2022GR0467). Informed consent was waived due to the retrospective nature of data collection and anonymization of patient identifiers prior to analysis.

## 3. Results

### 3.1. E. coli Resistance Trends

**Figure 1:**
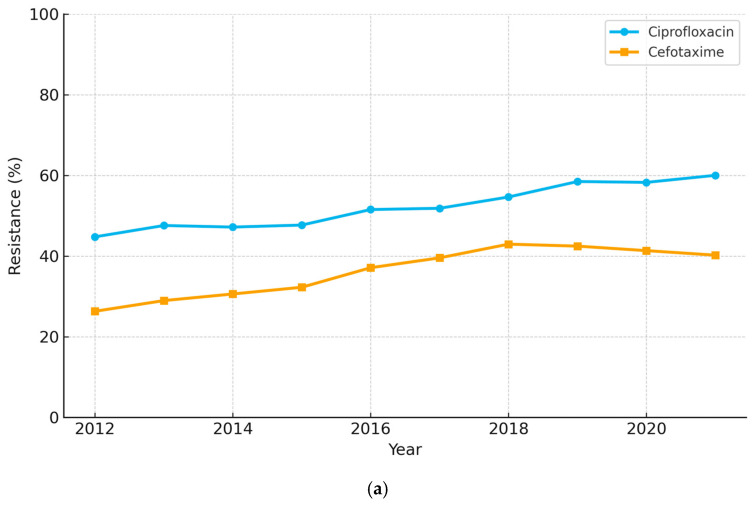

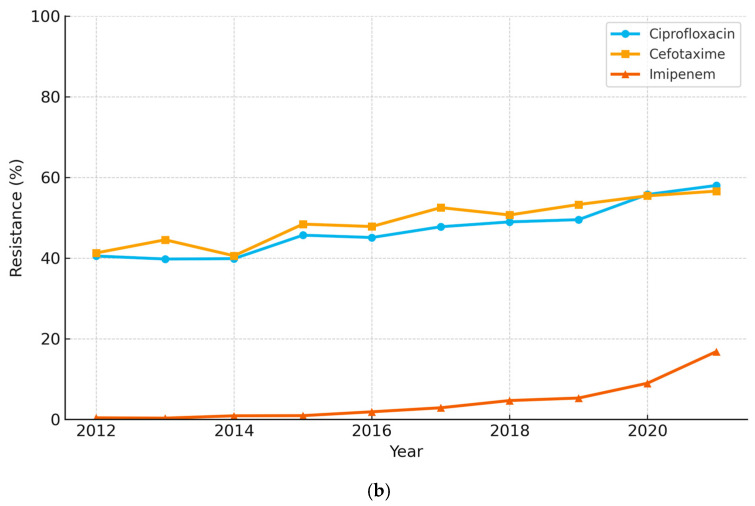
(**a**) Ten-year antimicrobial-resistance trends in urinary *Escherichia coli* (2012–2021). (**b**) Ten-year antimicrobial resistance trends in urinary *Klebsiella pneumoniae* (2012–2021).

Over the ten-year study period (2012–2021), *Escherichia coli* isolates showed significant increases in resistance to multiple antibiotics (Figure 1a; full numeric values are retained in Supplementary Table S1). Furthermore, Ciprofloxacin resistance rose from 44.8% to 60.0% (*p* < 0.001). Sensitivity analyses confirmed that the ascending trajectories for ciprofloxacin and cefotaxime in _*E. coli*_ persisted when (i) the COVID-19 years (2020–2021) were excluded and (ii) the earliest two years (2012–2013) were removed (both *p* ≤ 0.003; see Supplementary Table S4), while cefazolin (30.6% to 43.2%, *p* = 0.001) and cefotaxime (26.3% to 40.2%, *p* = 0.001) also demonstrated marked increases. Although amikacin and tigecycline started at relatively low resistance levels, both exhibited small but statistically significant upward trends (*p* = 0.042 each). Ampicillin, already high at 72.2% in 2012, rose slightly to 74.9% (*p* = 0.006), and aztreonam resistance increased from 24.3% to 31.2% (*p* = 0.005). Notably, carbapenem resistance also increased, with ertapenem rising from 0.3% to 1.2% (*p* = 0.006) and imipenem from 0.0% to 0.7% (*p* = 0.001). In contrast, no significant changes were observed for other tested agents, which generally remained stable or showed clinically minimal variations.

### 3.2. K. pneumoniae Resistance Trends

*Klebsiella pneumoniae* isolates (Figure 1b; detailed numbers are now provided in Supplementary Table S1) displayed similarly troubling trends. In particular, ertapenem and imipenem resistance increased from 3.0% to 18.1% (*p* = 0.001) and from 0.4% to 16.8% (*p* < 0.001), respectively, while tigecycline resistance rose from 12.5% to 30.9% (*p* = 0.006). Sensitivity analyses demonstrated that the upward trend for imipenem resistance in _*K. pneumoniae*_ remained significant in both restricted datasets (*p* ≤ 0.003; Supplementary Table S4). Among the broad-spectrum cephalosporins, resistance rates were already above 20% in 2012 and continued to climb for cefazolin (45.8% to 58.2%, *p* < 0.001), cefotaxime (41.3% to 56.6%, *p* < 0.001), aztreonam (41.1% to 54.7%, *p* < 0.001), and ceftazidime (42.2% to 53.5%, *p* < 0.001). Similar increases were also observed for ciprofloxacin (40.5% to 58.0%, *p* < 0.001), trimethoprim-sulfamethoxazole (28.9% to 49.0%, *p* < 0.001), amoxicillin-clavulanate (38.5% to 51.6%, *p* < 0.001), and piperacillin-tazobactam (34.3% to 45.8%, *p* = 0.013). By contrast, gentamicin varied between 15.6% and 30.4% without a clear upward trend, and amikacin resistance declined from 7.5% to 1.3% (*p* < 0.001).

### 3.3. Sensitivity Analysis Excluding 2020–2021 and 2012–2013

Removing the COVID-19 years or the earliest two study years did not alter the ascending trends (*p* < 0.005; Table S4a). The underlying annual percentages for 2012–2019 are listed in Table S4b, confirming that ciprofloxacin resistance in *E. coli* still rose from 44.8% to 58.5% and imipenem resistance in *K. pneumoniae* rose from 0.4% to 7.0%.

### 3.4. Hospital-Level Analysis

Hospital-specific analysis demonstrated marked heterogeneity (*E. coli* χ^2^ = 44.4 for ciprofloxacin, χ^2^ = 29.3 for cefotaxime; both *p* < 0.001; *K. pneumoniae* χ^2^ = 6.13 and 5.59, respectively; *p* = 0.047 and 0.061). In 2021, ciprofloxacin resistance was 61.5%/65.4%/54.8% in Hospitals A/B/C for *E. coli* and 60.5%/58.8%/54.4% for *K. pneumoniae*. Cefotaxime resistance showed a similar pattern—38.3%, 36.9%, 44.7% in *E. coli* versus 54.6%, 55.1%, 60.2% in *K. pneumoniae* (Supplementary Table S5).

### 3.5. ESBL-Producing E. coli and K. pneumoniae

**Figure 2:**
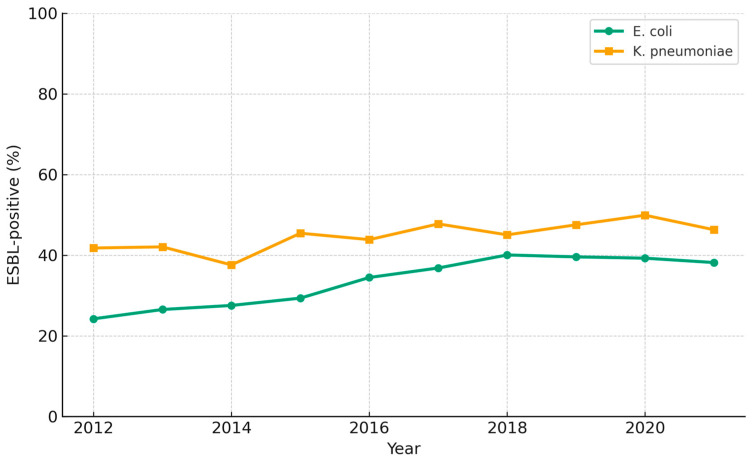
Ten-year trend (2012–2021) in the proportion of ESBL-producing *Escherichia coli* and *Klebsiella pneumoniae* among total isolates in tertiary-care hospitals across South Korea. Each data point represents the annual percentage of ESBL-positive isolates within each species, determined according to CLSI guidelines using third-generation cephalosporin susceptibility tests. *p*-values indicate the significance of the overall trend for *E. coli* (*p* = 0.003) and *K. pneumoniae* (*p* = 0.016) during the study period.

From 2012 to 2021, ESBL-producing *Escherichia coli* and *Klebsiella pneumoniae* increased from 24.1% to 38.2% and 39.2% to 46.4%, respectively (Figure 2). A concise ESBL-positive versus overall comparison for three sentinel antibiotics (2017, 2019, 2021) is provided in Table 1. From 2012 to 2021, the prevalence of ESBL-producing *E. coli* and *K. pneumoniae* increased from 24.1% to 38.2% and from 39.2% to 46.4%, respectively. The rates of *K. pneumoniae* remained stable over the last six years, and those of *E. coli* remained stable over the last three years, with both trends showing statistical significance (*E. coli*, *p* = 0.003; *K. pneumoniae*, *p* = 0.016). These data demonstrate a marked rise in ESBL-producing isolates of both organisms over the past decade.

**Figure 3:**
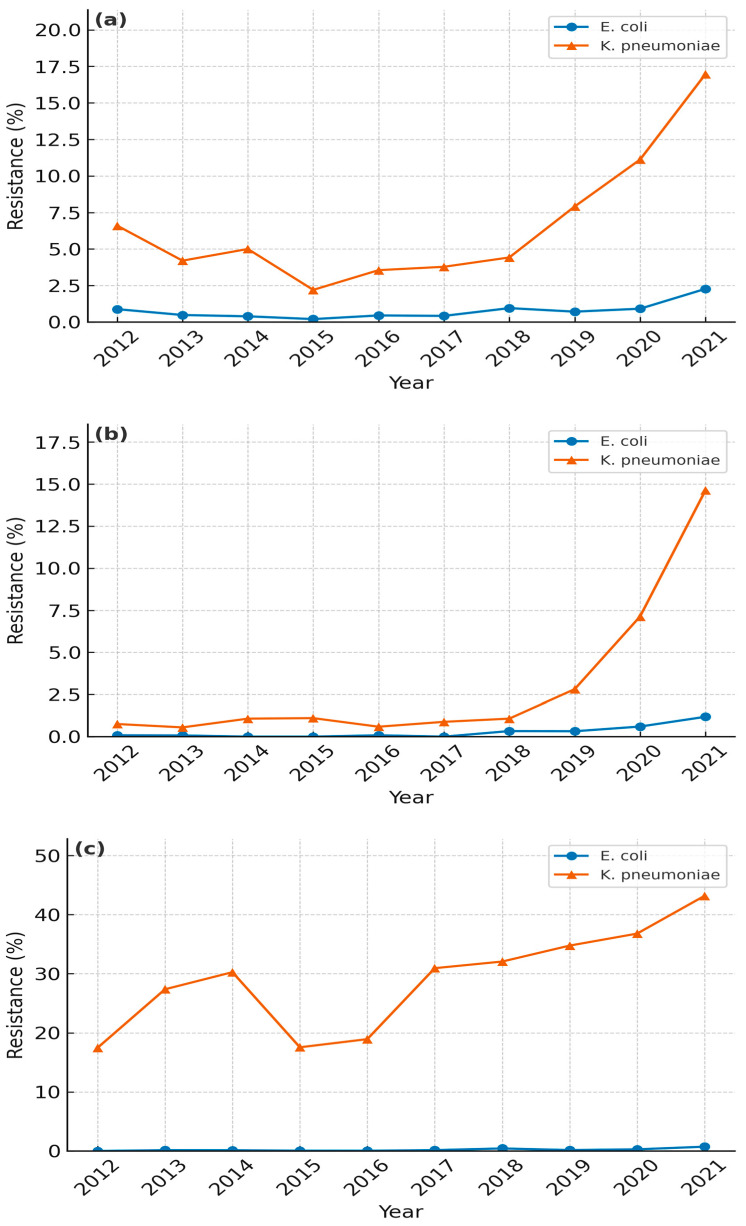
Annual resistance rates (%) of ESBL-producing *Escherichia coli* and *Klebsiella pneumoniae* from 2012 to 2021 for (**a**) Ertapenem, (**b**) Imipenem, and (**c**) Tigecycline. Exact percentages are listed in Supplementary Table S2.

Figure 3 shows annual resistance rates in ESBL-producing *E. coli* and *K. pneumoniae* or (a) ertapenem, (b) imipenem, and (c) tigecycline. Ertapenem resistance in *E. coli* rose from 0.9% to 2.3% (*p* = 0.098) and in *K. pneumoniae* from 6.6% to 17.0% (*p* = 0.128), although neither increase reached significance (Figure 3a). Imipenem resistance in *E. coli* increased modestly from 0.1% to 1.2% (*p* = 0.022), whereas in *K. pneumoniae,* the resistance rate jumped from 0.8% to 14.6% (*p* = 0.007), indicating a pronounced upward trend (Figure 3b). Tigecycline resistance remained very low in *E. coli* (0.0% to 0.7%, *p* = 0.002) but rose markedly in *K. pneumoniae* (17.5% to 43.2%, *p* < 0.001) (Figure 3c). Other antibiotics not illustrated in Figure 3 exhibited varied patterns (Table S2). In *E. coli*, resistance to ciprofloxacin (72.9% to 83.3%, *p* = 0.001), amoxicillin-clavulanate (47.8% to 43.9%, *p* = 0.025), cefepime (72.9% to 56.3%, *p* = 0.008), cefoxitin (35.7% to 23.7%, *p* < 0.001), and gentamicin (48.1% to 43.0%, *p* = 0.043) generally declined, whereas piperacillin-tazobactam remained relatively stable (15.8% to 13.2%, *p* = 0.31). In *K. pneumoniae*, amikacin resistance declined notably (from 14.9% to 1.3%, *p* < 0.001), while resistance to other agents fluctuated without a consistent trend. Overall, carbapenem and tigecycline resistance stayed relatively low yet increased slightly in ESBL-producing *E. coli*, whereas *K. pneumoniae* demonstrated a sharper rise, particularly for imipenem and tigecycline, underscoring the escalating clinical challenge posed by ESBL-producing *K. pneumoniae*.

## 4. Discussion

Our data reveal that ESBL-producing *K. pneumoniae* underwent a marked surge in carbapenem resistance from 2012 to 2018, reaching imipenem rates of 14.6% by the end of the study period (0.8% initially). Notably, while the proportion of ESBL-producing *K. pneumoniae* has stabilized over the last six years, carbapenem resistance within this subset has continued to climb, suggesting the ongoing acquisition of additional resistance mechanisms [16]. These organism-specific trajectories emerged in a domestic setting characterized by sustained high use of third-generation cephalosporins and fluoroquinolones and only partial enforcement of infection-control measures, factors explored later in this section. In contrast, ESBL-producing *E. coli* maintained relatively low carbapenem resistance (0.1% to 1.2%), indicating a divergent pattern between these two organisms. This local trend echoes a two-decade Korean surveillance study, in which imipenem resistance in *K. pneumoniae* increased steadily, while remaining negligible in *E. coli*, and carbapenem-nonsusceptible Enterobacteriaceae expanded ~1.5-fold annually [17].

Beyond the ESBL subset, overall *K. pneumoniae* likewise demonstrated a substantial increase in carbapenem resistance, with imipenem rising from 0.4% to 16.8%. This broader trend highlights that, although ESBL rates have plateaued, the entire *K. pneumoniae* population continues to evolve toward carbapenem resistance. These findings align with the European Centre for Disease Prevention and Control (ECDC) classification of carbapenem-resistant *K. pneumoniae* (CRKP) as a critical priority pathogen, highlighting the persistent threat of multidrug-resistant Enterobacteriaceae in both community- and hospital-onset urinary tract infections [18]. To contextualize our overall CRKP results internationally, the 2023 ECDC report (covering 2021 data) indicates that carbapenem resistance in *K. pneumoniae* ranges from under 1% in countries such as Sweden and the Netherlands to over 50% in Greece, with Italy and Romania hovering near 30%. Comparing these to the 2022 report (based on 2020 data) reveals incremental but consistent increases in most nations (e.g., Austria, from ~3.5% to 4.2%, and Greece, from ~45–50% to ~48–55%) [19,20]. Meanwhile, WHO GLASS 2022 shows that multiple countries in the Eastern Mediterranean (e.g., Egypt, from ~42% to ~46%) and Southeast Asia (e.g., India, from ~35% to 40%) have similarly documented an escalation of CRKP over the last two years [21]. Beyond the epidemiological figures, recent IDSA guidance summarizes that ~35% of U.S. CRE isolates already harbor carbapenemase genes (predominantly KPC and NDM), underscoring how plasmid-mediated mechanisms accelerate the international spread of resistance [22,23,24]. The timing and slope of our local CRKP curve parallels a 72% national increase in carbapenem consumption between 2015 and 2021 and the pandemic-related suspension of stewardship rounds in 2020–2021, suggesting policy and practice changes directly shape resistance trends. Although data completeness varies, these multi-year observations underscore a worldwide escalation of carbapenem-resistant *K. pneumoniae*, mirroring the ascending rates seen in our entire *K. pneumoniae* population. Such convergence reinforces the necessity of ongoing stewardship and timely de-escalation.

National Kor-GLASS data show that ciprofloxacin resistance among urinary *E. coli* isolates increased from 44.5% in 2016 to 45.0% in 2019 and remains ≥40% in each study year; cefotaxime resistance rose from 31.6% to 37.5% over the same period [25]. Similar trends are evident in *K. pneumoniae* (cefotaxime 39.7 → 41.8%) [25].

Our 2021 data show that urinary *E. coli* susceptibility to ciprofloxacin and cefotaxime is only 40% and 60%, respectively—well below the ≥80% threshold for blind empirical use [6,26]. Incorporating the 2023 KARMS survey [27], the latest international guidelines [22,26,28], we suggest: (i) uncomplicated cystitis—oral fosfomycin 3 g single dose or nitrofurantoin 100 mg twice daily × 5 days; (ii) community-onset complicated UTI without ESBL risk factors (prior ESBL isolate, recent ≥ 48 h IV antibiotics, urinary instrumentation, or long-term-care residence.)—single-dose amikacin 15 mg·kg^−1^ followed by culture-directed oral step-down (aminoglycoside 1-dose strategy endorsed in KAMR consensus) [27,29], (iii) complicated UTI with ESBL risk factors or early sepsis—empirical ertapenem 1 g daily, then de-escalate when susceptibility allows; (iv) healthcare-associated infection or septic shock—meropenem 1 g q8 h or imipenem-cilastatin 500 mg q6 h. Ceftazidime-avibactam may be used for documented carbapenemase-producing Enterobacterales, per Korean consensus [28]. Systematic de-escalation to the narrowest active agent (cefepime, TMP-SMX or a fluoroquinolone) is mandatory once culture results are available. These suggestions are appropriate for high-prevalence tertiary centers like ours but do not constitute a national standard; a prospective, multicenter outcomes study is required before establishing a universally applicable Korean guideline.

Several macro-level factors likely explain the persistent rise in fluoroquinolone and cephalosporin resistance. First, outpatient oral antibiotic consumption in Korea (26.1 DDD/1000 inhabitants/day in 2020) remains among the highest in the OECD [30,31]. Second, before the nationwide rollout of hospital antimicrobial stewardship programs (ASP) in 2021, only 38% of secondary-care facilities had formal restriction/audit policies (ASP coverage 38%). Third, apart from stewardship, universal active surveillance for ESBL/CRE colonization was confined to ICUs until late 2021; nursing-home and rehabilitation wards introduced admission screening only thereafter, leaving a sizeable reservoir for nosocomial spread. Fourth, COVID-19 led to greater empirical antibiotic use for febrile respiratory illness—a phenomenon quantified by a Korean nationwide cohort in which only 8% of 6871 COVID-19 admissions had culture-proven bacterial infection, yet 43% received anti-Pseudomonal or anti-MRSA agents [32,33]. A multi-country patient-level analysis likewise documented a significant pandemic-era surge in meropenem and piperacillin-tazobactam use in South Korean hospitals [34]. These observations highlight how crisis-driven prescribing can erode stewardship gains. Finally, the inter-facility transfer of chronically ill patients—often without universal screening for ESBL or carbapenem-resistant organisms—facilitates regional spread, as documented in Kor-GLASS sentinel sites. Collectively, the confluence of high antimicrobial consumption, delayed ASP enforcement, incomplete screening, and pandemic-related prescribing surges offers a plausible mechanistic explanation for the linear increases in ciprofloxacin, cefotaxime, and carbapenem resistance shown in Figure 1, Figure 2 and Figure 3.

Despite providing a decade-long perspective on urinary tract infection (UTI) pathogens, this study has several limitations that could be addressed for further enhancement. First, data were collected through 2021, so recent resistance trends from 2022 onward remain uncharacterized, which may affect the immediate clinical relevance of our findings. Second, ICD codes were used to identify UTIs, potentially overlooking patients coded under sepsis or septic shock, and we did not disaggregate infections into subtypes (e.g., cystitis vs. pyelonephritis). Third, focusing on two major uropathogens (*E. coli* and *K. pneumoniae*) might limit insights into other clinically essential bacteria. Finally, while the three participating tertiary hospitals captured diverse populations, these centers may not fully represent regional variations in antibiotic usage or resistance, and irreversible de-identification precluded ward-level (e.g., ICU) stratification; future OMOP-CDM linkage studies are planned to address this gap.

## 5. Conclusions

Our decade-long, multicenter analysis highlights the sustained burden of ESBL-producing *E. coli* and *K. pneumoniae*, with notable rises in carbapenem resistance among *K. pneumoniae*. High resistance rates to cephalosporins and fluoroquinolones further challenge conventional empirical regimens, particularly in complicated urinary tract infections (UTIs) or urosepsis. These results underscore the need for vigilant antimicrobial stewardship and tailored empirical therapy in Korea’s clinical practice, as well as broader surveillance to mitigate the spread of multidrug-resistant pathogens.

## Figures and Tables

**Table 1:** Comparative resistance rates (%) of ciprofloxacin, cefotaxime, and ertapenem for total versus ESBL-positive isolates of *Escherichia coli* and *Klebsiella pneumoniae* in 2017, 2019 and 2021.

Species	Year	Ciprofloxacin (Total)	Ciprofloxacin (ESBL+)	Cefotaxime (Total)	Cefotaxime (ESBL+)	Ertapenem (Total)	Ertapenem (ESBL+)
*E. coli*	2017	51.83	78.46	39.54	98.98	0.35	0.42
*E. coli*	2019	58.46	79.2	42.45	98.71	0.51	0.71
*E. coli*	2021	60	83.31	40.21	99.59	1.21	2.27
*K. pneumoniae*	2017	47.75	81.38	52.49	99.75	4.94	3.78
*K. pneumoniae*	2019	49.51	78.67	53.26	98.34	7.96	7.91
*K. pneumoniae*	2021	57.99	87.98	56.58	98.88	18.09	16.97

## Data Availability

The data presented in this study are available on reasonable request from the corresponding author, subject to approval by the Institutional Review Board.

## Supplementary Materials

The following supporting information can be downloaded at: https://www.mdpi.com/article/10.3390/diagnostics15162004/s1, Table S1: Yearly antimicrobial resistance rates (%) of *Escherichia coli* and *Klebsiella pneumoniae* isolates from 2012 to 2021; Table S2: Yearly antimicrobial resistance rates (%) of ESBL-producing *Escherichia coli* and *Klebsiella pneumoniae* isolates from 2012 to 2021; Table S3: Comparative resistance rates (%) of ciprofloxacin, cefotaxime and ertapenem for total versus ESBL-positive isolates of *Escherichia coli* and *Klebsiella pneumoniae* in 2017, 2019 and 2021; Table S4: (a) Cochran–Armitage trend tests for antimicrobial resistance in *Escherichia coli* and *Klebsiella pneumoniae* over the full period (2012–2021), pre-COVID (2012–2019), and trimmed period (2014–2021); (b) Pre COVID yearly resistance (%); Table S5. Hospital-specific 2021 resistance to ciprofloxacin (CIP) and cefotaxime (CTX, surrogate for ESBL production) in urinary *Escherichia coli* and *Klebsiella pneumoniae* from three tertiary-care centres.
