# Supplementary material for: Carbapenem Resistance and ESBL-Producing Enterobacteriaceae in Patients with Urological Infections from 2012 to 2021 in Three Korean Hospitals
Source: Diagnostics (Basel). 2025 Aug 11;15(16):2004. doi: 10.3390/diagnostics15162004 (PMC12385634; doi:10.3390/diagnostics15162004)
Supplement: Supplementary file 1 [file diagnostics-15-02004-s001.zip › diagnostics-3771449-supplementary.pdf]

Table S1. Yearly antimicrobial resistance rates (%) of Escherichia coli and Klebsiella pneumoniae isolates from 2012 to 2021

E.coli

Antimicrobial agent	2012	2013	2014	2015	2016	2017	2018	2019	2020	2021
Amikacin	0.67 (35/5232)	0.83 (45/5441)	0.64 (36/5622)	0.42 (28/6675)	0.67 (48/7126)	1.13 (81/7188)	1.23 (94/7632)	1.29 (101/7835)	0.98 (71/7271)	1.12 (72/6453)
Amoxicillin-clavulanate	26.59 (1390/5228)	30.06 (1634/5435)	31.31 (1758/5615)	31.3 (2086/6665)	29.25 (2081/7114)	29.06 (2086/7178)	31.09 (2371/7626)	29.57 (2313/7822)	30.56 (2220/7265)	28.3 (1826/6452)
Ampicillin	72.21 (3775/5228)	73.39 (3993/5441)	73.18 (4110/5616)	71.82 (4789/6668)	74.22 (5283/7118)	74.42 (5345/7182)	76.79 (5858/7629)	76.26 (5971/7830)	75.87 (5515/7269)	74.92 (4727/6309)
Ampicillin-sulbactam	14.29 (1/7)	80 (4/5)	42.86 (3/7)	35.71 (5/14)	53.33 (8/15)	61.53 (387/629)	62.01 (1513/2440)	59.79 (1292/2161)	54.05 (20/37)	86.11 (31/36)
Aztreonam	24.32 (1228/5050)	27.1 (1474/5440)	26.68 (1500/5622)	26.28 (1754/6674)	29.95 (2133/7122)	30.7 (2206/7186)	34.5 (2633/7632)	34.03 (2666/7835)	30.31 (2203/7268)	31.18 (2012/6453)
Cefazolin	30.57 (1010/3304)	32.81 (1784/5437)	33.71 (1893/5615)	35.63 (2375/6666)	40.24 (2863/7115)	41.98 (2757/6567)	45.85 (2384/5200)	45.94 (2606/5673)	44.38 (3212/7237)	43.16 (2772/6422)
Cefepime	17.64 (923/5232)	20.55 (1118/5441)	20.42 (1148/5622)	22.01 (1469/6675)	25.69 (1831/7126)	22.29 (1602/7188)	28.89 (2205/7632)	27.53 (2157/7836)	20.07 (1459/7271)	21.77 (1406/6457)
Cefotaxime	26.3 (1376/5232)	28.95 (1575/5441)	30.59 (1720/5622)	32.25 (2153/6675)	37.1 (2644/7126)	39.54 (2842/7188)	42.94 (3277/7632)	42.45 (3326/7836)	41.33 (3005/7270)	40.21 (2597/6458)
Cefoxitin	16.2 (847/5229)	17.9 (974/5441)	16.79 (943/5616)	17.19 (1146/6667)	17.07 (1215/7118)	16.27 (1169/7183)	17.23 (1314/7628)	15.64 (1225/7830)	15.49 (1126/7269)	15.01 (969/6455)
Ceftazidime	25.21 (1319/5232)	27.61 (1502/5441)	26.5 (1490/5622)	25.06 (1673/6675)	28.18 (2008/7126)	25.57 (1838/7188)	30.71 (2344/7632)	31.21 (2445/7835)	24.67 (1794/7271)	26.05 (1682/6458)
Ceftriaxone										
Ciprofloxacin	44.75 (1480/3307)	47.56 (2587/5439)	47.17 (2652/5622)	47.67 (3182/6675)	51.52 (3671/7126)	51.83 (3725/7187)	54.63 (4170/7633)	58.46 (4581/7836)	58.25 (4235/7271)	60 (3870/6450)
Ertapenem	0.27 (9/3290)	0.26 (14/5436)	0.18 (10/5614)	0.18 (12/6666)	0.18 (13/7114)	0.35 (25/7179)	0.46 (35/7628)	0.51 (40/7828)	0.63 (46/7261)	1.21 (78/6455)
Gentamicin	29.05 (1520/5232)	29.5 (1605/5441)	30.17 (1696/5622)	30.35 (2026/6675)	31.22 (2225/7126)	32.55 (2339/7186)	34.01 (2596/7632)	32.9 (2578/7835)	30.78 (2238/7270)	29.76 (1920/6451)
Imipenem	0.04 (2/5231)	0.04 (2/5440)	0 (0/5620)	0.03 (2/6673)	0.04 (3/7124)	0.1 (7/7185)	0.21 (16/7632)	0.37 (29/7836)	0.48 (35/7266)	0.7 (45/6458)
Levofloxacin	47.3 (912/1928)	100 (1/1)	25 (1/4)	83.33 (5/6)	50 (2/4)	53.29 (332/623)	57.14 (1393/2438)	55.46 (1198/2160)	44.44 (16/36)	71.88 (23/32)
Meropenem	0 (0/1931)	0 (0/3)	0 (0/8)	0 (0/13)	0 (0/14)	0.32 (2/625)	0.2 (5/2441)	0.42 (9/2164)	10.26 (4/39)	7.89 (3/38)
Piperacillin	69.86 (1349/1931)	57.14 (4/7)	87.5 (7/8)	66.67 (10/15)	62.5 (10/16)	72.89 (457/627)	72.93 (1778/2438)	72.12 (1560/2163)	62.5 (25/40)	88.89 (32/36)
Piperacillin-tazobactam	9.19 (435/4732)	9.24 (501/5423)	7.03 (394/5605)	8.04 (535/6657)	8.37 (594/7096)	7.69 (551/7162)	8.8 (671/7621)	7.92 (619/7813)	8.97 (646/7202)	7.55 (484/6407)
Tigecycline	0.09 (3/3305)	0.15 (8/5435)	0.09 (5/5620)	0.07 (5/6672)	0.06 (4/7122)	0.18 (13/7183)	0.37 (28/7631)	0.13 (10/7828)	0.23 (17/7265)	0.39 (25/6445)
Trimethoprim/ sulfamethoxazole	38.65 (2022/5232)	38.58 (2099/5441)	39.97 (2246/5619)	37.43 (2497/6672)	39.8 (2834/7121)	40.36 (2899/7182)	42.48 (3242/7631)	42.28 (3311/7832)	41.74 (3031/7262)	37.89 (2443/6448)

K.pn

Antimicrobial agent	2012	2013	2014	2015	2016	2017	2018	2019	2020	2021
Amikacin	7.48 (77/1030)	7.21 (94/1303)	3.55 (44/1240)	1.64 (23/1403)	2.91 (45/1544)	2.7 (45/1665)	1.72 (32/1863)	1.58 (32/2022)	1.04 (22/2107)	1.32 (28/2122)
Amoxicillin-clavulanate	38.45 (396/1030)	40.94 (533/1302)	36.77 (456/1240)	44.94 (631/1404)	41 (631/1539)	46.87 (779/1662)	46.22 (861/1863)	47.92 (969/2022)	52.09 (1097/2106)	51.63 (1096/2123)
Ampicillin	100 (1030/1030)	99.92 (1302/1303)	99.92 (1239/1240)	100 (1405/1405)	100 (1540/1540)	99.94 (1661/1662)	100 (1863/1863)	99.85 (2019/2022)	99.67 (2099/2106)	100 (2123/2123)
Aztreonam	41.07 (407/991)	43.36 (565/1303)	38.79 (481/1240)	46.87 (658/1404)	46.31 (715/1544)	49.37 (822/1665)	48.09 (896/1863)	49.8 (1007/2022)	52.09 (1098/2108)	54.73 (1162/2123)
Cefazolin	45.78 (320/699)	46.05 (600/1303)	41.85 (519/1240)	49.79 (699/1404)	50.26 (774/1540)	55.35 (869/1570)	53.99 (690/1278)	55.55 (796/1433)	57.58 (1204/2091)	58.15 (1213/2086)
Cefepime	28.64 (295/1030)	35 (456/1303)	31.94 (396/1240)	38.51 (541/1405)	40.8 (630/1544)	38.86 (647/1665)	40.58 (756/1863)	43.52 (880/2022)	42.55 (897/2108)	43.38 (921/2123)
Cefotaxime	41.26 (425/1030)	44.51 (580/1303)	40.55 (502/1238)	48.4 (680/1405)	47.8 (738/1544)	52.49 (874/1665)	50.67 (944/1863)	53.26 (1077/2022)	55.41 (1168/2108)	56.58 (1200/2121)
Cefoxitin	20.1 (207/1030)	20.64 (269/1303)	19.68 (244/1240)	18.93 (266/1405)	18.96 (292/1540)	26.29 (437/1662)	25.87 (482/1863)	25.17 (509/2022)	28.21 (594/2106)	28.26 (600/2123)
Ceftazidime	42.23 (435/1030)	44.67 (582/1303)	39.76 (493/1240)	46.41 (652/1405)	46.5 (718/1544)	47.99 (799/1665)	48.36 (901/1863)	50.45 (1020/2022)	50.28 (1060/2108)	53.51 (1136/2123)
Ciprofloxacin	40.49 (283/699)	39.75 (518/1303)	39.84 (494/1240)	45.66 (641/1404)	45.08 (696/1544)	47.75 (795/1665)	48.95 (912/1863)	49.51 (1001/2022)	55.74 (1175/2108)	57.99 (1230/2121)
Ertapenem	3.03 (21/692)	2 (26/1303)	2.92 (36/1232)	1.85 (26/1402)	3.84 (59/1536)	4.94 (82/1660)	6.44 (120/1862)	7.96 (161/2022)	11.16 (235/2106)	18.09 (384/2123)
Gentamicin	25.92 (267/1030)	22.87 (298/1303)	20 (248/1240)	15.61 (219/1403)	19.82 (306/1544)	30.39 (506/1665)	27.64 (515/1863)	25.82 (522/2022)	28.27 (596/2108)	23.33 (495/2122)
Imipenem	0.39 (4/1028)	0.31 (4/1303)	0.89 (11/1238)	0.93 (13/1404)	1.88 (29/1544)	2.88 (48/1665)	4.67 (87/1863)	5.29 (107/2022)	8.97 (189/2108)	16.82 (357/2123)
Piperacillin-tazobactam	34.29 (323/942)	30.85 (402/1303)	31.18 (386/1238)	40.23 (564/1402)	37.29 (575/1542)	38.61 (641/1660)	35.12 (654/1862)	35.85 (723/2017)	44.52 (935/2100)	45.78 (970/2119)
Tigecycline	12.45 (87/699)	14.98 (195/1302)	14.19 (176/1240)	11.47 (161/1404)	11.41 (176/1543)	18.26 (304/1665)	21.04 (392/1863)	22.65 (458/2022)	23.67 (499/2108)	30.85 (654/2120)
Trimethoprim/ sulfamethoxazole	28.93 (298/1030)	34.64 (451/1302)	30.89 (383/1240)	34.47 (484/1404)	37.56 (580/1544)	45.13 (751/1664)	43.64 (813/1863)	48.07 (972/2022)	47.94 (1010/2107)	48.99 (1039/2121)

Table S2. Yearly antimicrobial resistance rates (%) of ESBL-producing Escherichia coli and Klebsiella pneumoniae isolates from 2012 to 2021

E.coli

Antimicrobial agent	2012	2013	2014	2015	2016	2017	2018	2019	2020	2021
Amikacin	2.62 (33/1260)	2.7 (39/1445)	1.74 (27/1548)	1.02 (20/1959)	1.55 (38/2456)	2.15 (57/2646)	2.58 (79/3057)	2.48 (77/3101)	1.72 (49/2854)	2.19 (54/2463)
Amoxicillin-clavulanate	47.78 (602/1260)	51.97 (751/1445)	54.46 (843/1548)	53.09 (1040/1959)	45.62 (1120/2455)	45.9 (1214/2645)	46.22 (1413/3057)	42.89 (1330/3101)	47.2 (1347/2854)	43.94 (1084/2467)
Ampicillin	100 (1260/1260)	100 (1445/1445)	99.81 (1545/1548)	99.85 (1956/1959)	99.92 (2454/2456)	99.96 (2644/2645)	99.61 (3045/3057)	99.61 (3089/3101)	99.89 (2851/2854)	100 (2467/2467)
Aztreonam	99.17 (1195/1205)	99.38 (1435/1444)	93.35 (1445/1548)	85.34 (1671/1958)	84.58 (2074/2452)	79.61 (2105/2644)	82.14 (2511/3057)	82.1 (2545/3100)	75.24 (2145/2851)	79.99 (1971/2464)
Cefazolin	99.75 (799/801)	99.72 (1441/1445)	99.55 (1541/1548)	99.34 (1946/1959)	98.53 (2419/2455)	99.21 (2397/2416)	99.28 (2059/2074)	99.47 (2248/2260)	99.3 (2826/2846)	99.96 (2444/2445)
Cefepime	72.94 (919/1260)	77.16 (1115/1445)	73.32 (1135/1548)	73.86 (1447/1959)	74.02 (1818/2456)	59.07 (1563/2646)	70.56 (2157/3057)	67.88 (2105/3101)	50.32 (1436/2854)	56.34 (1390/2467)
Cefotaxime	99.13 (1249/1260)	99.52 (1438/1445)	99.22 (1536/1548)	98.83 (1936/1959)	98 (2407/2456)	98.98 (2619/2646)	98.36 (3007/3057)	98.71 (3061/3101)	99.19 (2830/2853)	99.59 (2457/2467)
Cefoxitin	35.71 (450/1260)	35.64 (515/1445)	32.3 (500/1548)	32.82 (643/1959)	29.11 (715/2456)	25.36 (671/2646)	25.09 (767/3057)	22.25 (690/3101)	24.15 (689/2853)	23.68 (584/2466)
Ceftazidime	98.89 (1246/1260)	99.24 (1434/1445)	89.34 (1383/1548)	78.1 (1530/1959)	76.55 (1880/2456)	64.36 (1703/2646)	71.25 (2178/3057)	72.23 (2239/3100)	58.69 (1675/2854)	64.86 (1600/2467)
Ciprofloxacin	72.91 (584/801)	74.29 (1072/1443)	76.49 (1184/1548)	77.39 (1516/1959)	80.58 (1979/2456)	78.46 (2076/2646)	77.85 (2380/3057)	79.2 (2456/3101)	80.31 (2292/2854)	83.31 (2051/2462)
Ertapenem	0.88 (7/799)	0.48 (7/1444)	0.39 (6/1548)	0.2 (4/1959)	0.45 (11/2455)	0.42 (11/2644)	0.95 (29/3057)	0.71 (22/3101)	0.91 (26/2848)	2.27 (56/2467)
Gentamicin	48.1 (606/1260)	47.2 (682/1445)	45.99 (712/1548)	49.92 (978/1959)	47.64 (1170/2456)	48.47 (1282/2645)	47.01 (1437/3057)	43.87 (1360/3100)	42.71 (1219/2854)	42.97 (1058/2462)
Imipenem	0.08 (1/1260)	0.07 (1/1444)	0 (0/1548)	0 (0/1959)	0.08 (2/2454)	0 (0/2644)	0.33 (10/3056)	0.32 (10/3101)	0.6 (17/2853)	1.18 (29/2467)
Piperacillin-tazobactam	15.79 (181/1146)	16.26 (233/1433)	11.65 (180/1545)	14.16 (277/1956)	13.1 (320/2443)	11.63 (307/2640)	14.78 (451/3052)	11.56 (358/3096)	15.01 (424/2825)	13.21 (324/2453)
Tigecycline	0 (0/801)	0.14 (2/1445)	0.13 (2/1548)	0.05 (1/1959)	0.04 (1/2455)	0.15 (4/2645)	0.43 (13/3057)	0.16 (5/3101)	0.28 (8/2854)	0.73 (18/2461)
Trimethoprim/ sulfamethoxazole	59.05 (744/1260)	55.22 (798/1445)	56.65 (877/1548)	55.64 (1090/1959)	55.91 (1372/2454)	54.39 (1437/2642)	56.68 (1732/3056)	56.94 (1765/3100)	56.32 (1605/2850)	52.42 (1290/2461)

K.pneumoniae

Antimicrobial agent	2012	2013	2014	2015	2016	2017	2018	2019	2020	2021
Amikacin	14.85 (60/404)	14.05 (77/548)	7.08 (33/466)	3.14 (20/637)	5.02 (34/677)	3.52 (28/795)	2.86 (24/839)	2.39 (23/961)	0.86 (9/1052)	1.32 (13/983)
Amoxicillin-clavulanate	79.21 (320/404)	83.58 (458/548)	79.61 (371/466)	83.86 (535/638)	75.59 (511/676)	78.74 (626/795)	77.12 (647/839)	77.94 (749/961)	83.46 (878/1052)	80.69 (794/984)
Ampicillin	100 (404/404)	100 (548/548)	100 (466/466)	100 (638/638)	100 (677/677)	100 (795/795)	100 (839/839)	100 (961/961)	99.81 (1050/1052)	100 (984/984)
Aztreonam	99.75 (392/393)	99.45 (545/548)	97 (452/466)	97.18 (620/638)	96.9 (656/677)	96.1 (764/795)	94.04 (789/839)	93.96 (903/961)	93.25 (981/1052)	95.83 (943/984)

Cefazolin	100 (292/292)	100 (548/548)	100 (466/466)	99.53 (635/638)	99.7 (675/677)	99.87 (750/751)	98.78 (569/576)	99.12 (675/681)	98.95 (1035/1046)	99.69 (960/963)
Cefepime	72.77 (294/404)	82.12 (450/548)	83.05 (387/466)	82.45 (526/638)	86.56 (586/677)	74.72 (594/795)	80.33 (674/839)	82.31 (791/961)	75.1 (790/1052)	74.09 (729/984)
Cefotaxime	99.75 (403/404)	99.64 (546/548)	100 (464/464)	99.69 (636/638)	98.67 (668/677)	99.75 (793/795)	97.97 (822/839)	98.34 (945/961)	98.57 (1037/1052)	98.88 (971/982)
Cefoxitin	31.19 (126/404)	35.04 (192/548)	35.41 (165/466)	27.12 (173/638)	27.18 (184/677)	37.86 (301/795)	34.09 (286/839)	32.99 (317/961)	37.55 (395/1052)	33.74 (332/984)
Ceftazidime	99.75 (403/404)	99.45 (545/548)	97.21 (453/466)	94.36 (602/638)	94.83 (642/677)	90.69 (721/795)	92.13 (773/839)	92.61 (890/961)	88.4 (930/1052)	92.17 (907/984)
Ciprofloxacin	76.71 (224/292)	79.56 (436/548)	84.55 (394/466)	83.86 (535/638)	81.83 (554/677)	81.38 (647/795)	78.9 (662/839)	78.67 (756/961)	84.41 (888/1052)	87.98 (864/982)
Ertapenem	6.6 (19/288)	4.2 (23/548)	5 (23/460)	2.2 (14/637)	3.55 (24/677)	3.78 (30/793)	4.42 (37/838)	7.91 (76/961)	11.12 (117/1052)	16.97 (167/984)
Gentamicin	54.7 (221/404)	46.53 (255/548)	43.35 (202/466)	30.82 (196/636)	39 (264/677)	55.85 (444/795)	48.99 (411/839)	45.47 (437/961)	48.57 (511/1052)	38.15 (375/983)
Imipenem	0.75 (3/402)	0.55 (3/548)	1.07 (5/466)	1.1 (7/638)	0.59 (4/677)	0.88 (7/795)	1.07 (9/839)	2.81 (27/961)	7.13 (75/1052)	14.63 (144/984)
Piperacillin-tazobactam	73.58 (273/371)	64.05 (351/548)	71.4 (332/465)	76.45 (487/637)	68.69 (465/677)	66.46 (527/793)	57.21 (480/839)	58.92 (565/959)	71.69 (752/1049)	71.59 (703/982)
Tigecycline	17.47 (51/292)	27.37 (150/548)	30.26 (141/466)	17.55 (112/638)	18.93 (128/676)	30.94 (246/795)	32.06 (269/839)	34.76 (334/961)	36.79 (387/1052)	43.18 (424/982)
Trimethoprim/sulfamethoxazole	56.19 (227/404)	64.23 (352/548)	60.94 (284/466)	64.84 (413/637)	69.57 (471/677)	76.32 (606/794)	73.54 (617/839)	80.65 (775/961)	73.07 (768/1051)	73.93 (726/982)

Table S3. Comparative resistance rates (%) of ciprofloxacin, cefotaxime and ertapenem for total versus ESBL-positive isolates of *Escherichia coli* and *Klebsiella pneumoniae* in 2017, 2019 and 2021

Species	Year	Ciprofloxacin (Total)	Ciprofloxacin (ESBL+)	Cefotaxime (Total)	Cefotaxime (ESBL+)	Ertapenem (Total)	Ertapenem (ESBL+)
E. coli	2017	51.83	78.46	39.54	98.98	0.35	0.42
E. coli	2019	58.46	79.2	42.45	98.71	0.51	0.71
E. coli	2021	60	83.31	40.21	99.59	1.21	2.27
K. pneumoniae	2017	47.75	81.38	52.49	99.75	4.94	3.78
K. pneumoniae	2019	49.51	78.67	53.26	98.34	7.96	7.91
K. pneumoniae	2021	57.99	87.98	56.58	98.88	18.09	16.97

Table S4a. Cochran–Armitage trend tests for antimicrobial resistance in *Escherichia coli* and *Klebsiella pneumoniae* over the full period (2012–2021), pre-COVID (2012–2019), and trimmed period (2014–2021)

**Species**	**Antibiotic**	**Full period p-value (2012-2021)**	**Pre-COVID p-value (2012-2019)**	**Trimmed period p-value (2014-2021)**
E. coli	Ciprofloxacin	<0.001	<0.001	<0.001
E. coli	Cefotaxime	0.001	0.002	0.002
K. pneumoniae	Imipenem	0.001	0.003	0.003

Table S4b – Pre-COVID yearly resistance (%)

Year	E. coli Ciprofloxacin (%)	E. coli Cefotaxime (%)	K. pneumoniae Imipenem (%)
2012	44.75	26.3	0.39
2013	47.56	28.95	0.31
2014	47.17	30.59	0.89
2015	47.67	32.25	0.93
2016	51.52	37.1	1.88
2017	51.83	39.54	2.88
2018	54.63	42.94	4.67
2019	58.46	42.45	5.29

Table S5. Hospital-specific 2021 resistance to ciprofloxacin (CIP) and cefotaxime (CTX, surrogate for ESBL production) in urinary *Escherichia coli* and *Klebsiella pneumoniae* from three tertiary-care centres.

Hospital	*E.coli*			*K. pneumoniae*		
	Total isolates (n)	CIP-R n (%)	CTX-R n (%)	Total isolates (n)	CIP-R n (%)	CTX-R n (%)
A	2902	1785(61.5)	1112(38.3)	850	514 (60.5 %)	464 (54.6 %)
B	1340	876(65.4)	494(36.9)	572	336 (58.8 %)	315 (55.1 %)
C	2216	1213(54.8)	991(44.7)	703	382 (54.4 %)	423 (60.2 %)
χ^2 (2 df)		44.4	29.3		6.13	5.59
p value		<0.001	<0.001		0.047	0.061
